# Early‐stage evaluation of the Treehouse4Two Retreat Program to support people living with dementia and carer dyads living in rural Victoria, Australia

**DOI:** 10.1002/alz70858_098481

**Published:** 2025-12-25

**Authors:** Shahinoor Akter, Tshepo Rasekaba, Phil Catterson, Sean MacDermott

**Affiliations:** ^1^ Care Economy Research Institute, La Trobe University, Albury‐Wodonga, VIC, Australia; ^2^ John Richards Centre for Rural Ageing Research, La Trobe Rural Health School, La Trobe University, Albury‐Wodonga, VIC, Australia; ^3^ Central Highlands Rural Health, Daylesford, VIC, Australia

## Abstract

**Background:**

Innovative respite services are crucial for improving the quality of life (QoL) of people living with dementia and their carers, particularly in rural areas where resources are limited. The Central Highlands Rural Health implemented an innovative Treehouse4Two Retreat Program (T4Two) in rural Victoria, Australia in July 2023 with an aim to improve QoL for people living with dementia, ease caregiving burdens, and promote positive respite experiences. The Program is a 3‐day retreat and respite for people living with dementia and carer dyads. This abstract presents early evaluation findings of the Program to assess its initial acceptability and its impacts on the dyads.

**Methods:**

A mixed methods approach was used to collect, analyze, and interpret primary qualitative and secondary quantitative data, informed by the RE‐AIM (Reach, Efficacy, Adoption, Implementation, and Maintenance) framework. A total of 29 dyads participated in five retreats between August 2023 and February 2024. The qualitative data were collected through 9 dyadic interviews and a focus group discussion with program staff. Secondary survey data on quality of life (QoL‐AD), care burden (Zarit Burden Interview (ZBI)) and satisfaction with the program were included for analysis. Qualitative data were analysed thematically while descriptive analysis was conducted for quantitative data. Free‐text survey responses were analysed through content analysis.

**Results:**

Findings indicated that the Program reached the community beyond its service catchment areas, demonstrating a strong demand for respite services. Participants reported that this Program effectively addressed the support gaps they encountered and allowed them to access necessary resources sooner. Carer participants felt that education sessions substantially improved their dementia knowledge and boosted their confidence as carer. While the QoL‐AD and ZBI scores were not statistically significant—likely due to the small sample size and incomplete surveys – the satisfaction data indicated a high level of satisfaction among the participants.

**Conclusions:**

While the Program activities are ongoing, the early‐stage evaluation findings of the T4Two Program shows that it not only effectively addresses gaps in respite care in resources‐poor areas but also showing potential for scalability and sustainability which could significantly improve QoL for those affected by dementia.

